# Factors influencing the variation in GMS prescribing expenditure in Ireland

**DOI:** 10.1186/s13561-016-0090-x

**Published:** 2016-03-29

**Authors:** A. ConwayLenihan, S. Ahern, S. Moore, J. Cronin, N. Woods

**Affiliations:** 1 Department of Management & Enterprise, Cork Institute of Technology, Rossa Avenue, Bishopstown Cork, Ireland; 2 Centre for Policy Studies, University College Cork, 6 Bloomfield Terrace, Western Road, Cork, Ireland

## Abstract

**Background:**

Pharmaceutical expenditure growth is a familiar feature in many Western health systems and is a real concern for policymakers. A state funded General Medical Services (GMS) scheme in Ireland experienced an increase in prescription expenditure of 414 % between 1998 and 2012. This paper seeks to explore the rationale for this growth by investigating the composition (Anatomical Therapeutic Chemical (ATC) Group level 1 & 5) and drivers of GMS drug expenditure in Ireland in 2012.

**Methods:**

A cross-sectional study was carried out on the Health Service Executive-Primary Care Reimbursement Service (HSE-PCRS) population prescribing database (*n* = 1,630,775). Three models were applied to test the association between annual expenditure per claimant whilst controlling for age, sex, region, and the pharmacology of the drugs as represented by the main ATC groups.

**Results:**

The mean annual cost per claimant was €751 (median = €211; SD = €1323.10; range = €3.27–€298,670). Age, sex, and regions were all significant contributory factors of expenditure, with gender having the greatest impact (β = 0.107). Those aged over 75 (β =1.195) were the greatest contributors to annual GMS prescribing costs. As regards regions, the South has the greatest cost increasing impact. When the ATC groups were included the impact of gender is diluted by the pharmacology of the products, with cardiovascular prescribing (ATC ‘C’) most influential (β = 1.229) and the explanatory power of the model increased from 40 % to 60 %.

**Conclusion:**

Whilst policies aimed at cost containment (co-payment charges; generic substitution; reference pricing; adjustments to GMS eligibility) can be used to curtail expenditure, health promotional programs and educational interventions should be given equal emphasis. Also policies intended to affect physicians’ prescribing behaviour include guidelines, information (about price and less expensive alternatives) and feedback, and the use of budgetary restrictions could yield savings in Ireland and can be easily translated to the international context.

## Background

Pharmaceutical expenditure is a considerable and growing cost to health care systems in Western European Union (EU) countries, including Ireland. Consequently, it is important to identify the factors influencing this rise in expenditure so as to ensure the sustainability of health systems going forward. Information on pharmaceutical expenditure trends is necessary to facilitate informed decision making about efficient resource allocation, optimum budget allocation, and future policy decisions.

Pharmaceutical expenditure growth is not a recent phenomenon as it has been growing faster than Gross Domestic Product (GDP) in all European countries since the 1980s [1]. A systematic review of the factors influencing pharmaceutical expenditure identified that drug utilisation; drug therapies; price; and new drugs; are the main drivers of pharmaceutical expenditure [2], and population growth and aging have been found to be additional factors in Ireland [3]. In 2012, Ireland spent 14 % of total government expenditure on health which is comparable with the EU average (EU 28 average). Irish health spending accounted for 8.9 % of GDP, just above the EU28 average of 8.7 % [4].

Approximately 14 % of public health expenditure in Ireland was spent on pharmaceuticals in 2012 [5]. Irish pharmaceutical expenditure per capita relative to other Organisation for Economic Co-Operation and Development (OECD) countries increased significantly from 20th highest of 27 countries in 2000 to 3rd highest of 25 countries in 2010 [6]. In 2012, the state accounted for €2.6 billion (19 %) of overall health expenditure (€13.71bn) in Ireland through its publicly funded Community Drug Schemes (CDS). There are eleven CDS schemes. The General Medical Services (GMS) scheme accounted for €1.28 billion, 50 % of community drug expenditure, the High Tech Drugs scheme accounted for 385 million (15 %) and the Drug Payments scheme accounted for 126 million (5 %). This paper focuses on the GMS scheme, the largest community drug scheme [7].

The most significant contributor to pharmaceutical expenditure in Ireland is the GMS (medical card) scheme. This is a community drug scheme, whereby drugs, medicines and appliances supplied under the Scheme are provided through retail pharmacies. All GMS claims are processed and paid by the Primary Care Reimbursement Service (PCRS). The scheme is means tested and eligibility is based on income. Persons who are affected by certain medical conditions are also eligible for the scheme. Those who are unable without undue hardship to arrange general practitioner medical and surgical services for themselves and their dependants are eligible to receive free general medical service under the scheme and are issued with a medical card. The scheme is financed by the state with a co-payment from each eligible person introduced in 2010. Since October 2010, each person on the scheme has incurred a €0.50 charge for every prescription item dispensed. This was subsequently increased to €1.50 in 2013 and is currently €2.50 per prescription item up to a maximum of €25 per family per month. Between July 2001 and December 2008, all persons over 70 were granted a free medical card. Due to the economic crisis, eligibility criteria were introduced on the 1^st^ of January 2009 so that all persons ≥70 were means tested.

**Fig. 1:**
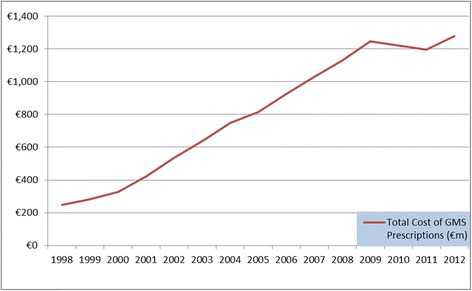
Total Cost (€m) of GMS Prescriptions between 1998 and 2012

Since 2000 GMS expenditure on pharmaceuticals has been rising rapidly, as illustrated in Fig. 1. The government introduced cost containment measures during the economic crisis to combat this trend such as co-payment charges for claimants,^1^ adjustments to GMS eligibility criteria, reference pricing and the promotion of generic substitution [8]. Despite these measures, Irish pharmaceutical spending per capita in 2012 was the third highest amongst EU countries at €500, 40 % above the EU average (€350) [4]. The total cost of GMS prescriptions has increased from €249 million in 1998 to €1.24 billion in 2009 (400 % increase) as illustrated in Fig. 1. GMS expenditure accounted for 9 % of the overall health budget in 2012 [9].

The total cost of GMS prescriptions decreased in 2010 and 2011, due to government cut backs and the introduction in cost containment measures, before increasing again to €1.28 billion in 2012, at a time when GMS coverage rates^2^ increased from 35 % of the population in 1998 to over 40 % in 2012. A study of projected GMS costs for Ireland found that GMS expenditure could rise to €2.5 billion by 2026 [10].

Between 1998 and 2012, between 67 % and 70 % of total annual GMS expenditure has been accounted for by four of the Anatomical Therapeutic Chemical (ATC) groups; Nervous (N) system, Cardiovascular (C) system, Alimentary Tract and Metabolism (A) system and Respiratory (R) system (Annual (PCRS) Reports, 1998–2012). In 2012, €872 million of total GMS expenditure (€1288.82 million) was recorded across the four ATC groups equating to 68 % of GMS expenditure [7]. Table 1 reports the findings of previous studies.

**Table 1:** Previous Studies

Country	Author	Findings
British Colombia (2006)	Morgan [24]	A population study on drug expenditure in 1996 and 2002 found 75 % of total drug costs were accounted for by 5 ATC groups in descending order of expenditure, ‘C’, ‘N’, ‘A’, ‘G’ (Genito Urinary System & Sex Hormones) and ‘J’ (Anti-infectives for Systemic Use).
Sweden (1998)	Gerdtham et al. [34]	This study identified ATC group ‘N’ contributed the most to drug expenditure.
Sweden (2004)	Gerdtham & Lundin [35]	This study identified ATC groups ‘N’, ‘C’ ‘and ‘A’ contributed the most to drug expenditure.
Sweden (2003)	Klarin et al. [36]	A study carried out on an elderly population (84+) found ‘C’, ‘N’ and ‘A’ were the most commonly prescribed drugs.
Spain (2011)	Vivas et al. [13]	This study reported the cost of drugs used to treat hyperlipidemia (from ATC ‘C’), respiratory illnesses, asthma (from ATC ‘R’) and diabetes (from ATC ‘A’) represents 37 % of total pharmaceutical expenditure on chronic conditions in Valencia.
Ireland (2006)	Naughton et al. [21]	A study based on an elderly Irish population (≥70 years) found that Cardiovascular Disease (CVD) is the most prevalent condition across all regions using a 2006 PCRS pharmacy database.
Various Studies	[3, 10, 20, 26, 30, 37]	A number of studies have identified age as a factor influencing pharmaceutical spending.
United States (1997)	Mueller et al. [30]	A study in the United States found the elderly (65+) accounted for 34 % of total pharmaceutical expenditure, children (≤17) accounted for 9 %, and those aged (18–64) accounted for the remaining 57 %. Of total drug expenditure, 41 % was spent on cardiovascular and renal drugs and 14 % on respiratory tract drugs.
3 European Countries (2008)	Sturkenboom et al. [20]	This study found that respiratory drugs were the most prescribed drugs and cardiovascular drugs were the least prescribed drugs for children (≤18).
Spain (2008)	Fernandez et al. [26]	A study in Catalonia, investigated how age influenced drug utilisation and found drug use was highest among infants (0–4) and those aged 55 and older.
Ireland (2015)	Conway et al. [10]	A recent study identified children (≤11) and the over 65 s as the two key drivers of future GMS costs in Ireland.
Various Studies	[2, 13, 23, 37]	Several studies have reported that females are more likely to utilise drugs than males.
United States (2000)	Steinberg et al. [37]	Males aged 65 and over were 50 % more likely not to be prescribed any drugs.
Sweden (2013)	Loikas et al. [23]	This study examined gender differences in drug prescribing and found 59 % of men and 76 % of women were dispensed at least one drug. Females were found to be prescribed more nervous drugs and males more lipid lowering (cardiovascular) drugs.
Various Studies	[12, 14, 15]	Geographical location is also considered in international studies as a predictor of pharmaceutical expenditure.
United States (2003)	Wrobel et al. [12]	A Medicare study examined the predictability of drug expenditure and found demographic variables (age, sex, disability & geographic location), explained only 5 % of the variation in drug expenditure. The inclusion of health status measures improved the explanatory power considerably.

The objective of this research is to investigate the significant drivers of GMS expenditure whilst controlling for age, gender, regional disparities, and the main therapeutic use of the main active ingredient of medicinal products i.e., ATC groups. The main contribution of this paper will identify the ATC groups by gender, age and region generating the highest level of cost. It will identify the ATC Level 5 i.e., specific drug within the ATC group that are generating the highest cost. We make recommendations on what policies that can be introduced to reduce cost and generate savings. These policies that will reduce cost can be translated internationally and would yield savings in the international context.

## Methods

### Study design and data

A cross-sectional study was carried out on the Irish Health Service Executive (HSE)-PCRS pharmacy claims database for the year 2012 (*n* = 1.63 million claimants). Each GMS claimant is identified by a unique dispense number. There are approximately 60 million observations in the database and the data was aggregated to create one observation per claimant. There are 40 variables in the database, of which seven are included in this research. These include demographic information (age, sex, region), cost data (ingredient cost, Value Added Tax (VAT), dispensing fees) and ATC group. For the purpose of this analysis, total annual cost is the sum of ingredient cost, VAT and dispensing fees for 2012. The pharmacology is coded using the World Health Organisation (WHO) ATC classification system^3^ [11]. The analysis of the pharmacology is undertaken at the 1st level of the ATC classification system.

Modelling health and drug expenditure varies internationally [2]. A number of studies have used Ordinary Least Squares (OLS) to model the variation in drug and health costs [12–18]. Following a review of the literature and expert opinion, the OLS regression model was deemed the most appropriate model to use.

### Empirical analysis

Three models were developed to investigate the association between the total annual GMS cost per claimant and the independent variables. The cost data is positively skewed (skewness = 16.140) and a log transformation was applied to normalise the data. This approach has been previously used [17, 19]. In the first model we estimate the impact on the log of annual total cost per claimant using OLS regression whilst controlling for age, sex, and the 4 HSE regions (Dublin Mid-Leinster (ML), Dublin North East (NE), South and West). The square and cube of age were considered as age has a non-linear relationship with the log of total cost. The inclusion of these two variables does not add to the explanatory power of the model and were excluded.

In the first model we estimate the impact of age, sex and region on the log of total cost.

$$ LogTC = {\beta}_0+{\beta}_1{X}_1 + {\beta}_2{X}_2 + {\beta}_3{X}_3+\dots + {\beta}_5{X}_5 + \varepsilon $$

where β_0_ is the constant, X_1_ = age, *X*_2_ = female, X_3_ = Dublin ML, X_4_ = West and X_5_ = South, (Reference category = Dublin NE, Male).

In the second model in addition to the demographic variables, we estimate the impact of pharmacology on the log of total cost by including the main ATC groups.

$$ LogTC = {\beta}_0+{\beta}_1{X}_1 + {\beta}_2{X}_2 + {\beta}_3{X}_3+\dots + {\beta}_8{X}_8 + \varepsilon $$

where X_1_ = age, *X*_2_ = female, X_3_ = Dublin ML, X_4_ = West, X_5_ = South, X_6_ = ‘A’ ATC group, X_7_ = ‘C’ ATC group and X_8_ = ‘N’ ATC group (Reference category = Dublin NE, ‘R’ ATC, Male).

In the third model in addition to the demographic and pharmacological variables, we estimate the model using 11 age categories, to ascertain the impact of age categories on cost.

$$ LogTC = {\beta}_0+{\beta}_1{X}_1 + {\beta}_2{X}_2 + {\beta}_3{X}_3+\dots + {\beta}_{17}{X}_{17} + \varepsilon $$

where X_1_ = female, *X*_2_ = Dublin ML, X_3_ = West, X_4_ = South, X_5_ = ‘A’ ATC group, X_6_ = ‘C’ ATC group and X_7_ = ‘N’ ATC group, plus 10 dummy variables for age cohort (X_8_-X_17_). (Reference category Age = 12–15, Dublin NE, ‘R’ ATC group, Male)

The models were estimated by OLS regression. Each model was tested for normality, homoscedasticity and linearity. The Variance Inflation Factor (VIF) and tolerance factors were obtained to test for multicollinearity. All statistical tests were two-sided at the 5 % significance level. Aggregation by GMS claimant was carried out in R Studio version 2.15.3. Statistical Analysis was performed using SPSS 22.

## Results

### Statistical analysis

The mean annual cost per claimant was €751 (median = €211; SD = €1323.10; range = €3.27–€298,670). As regards age, 14 % were 75 or over, with 4 % between 12 and 15. Over 55 % of claimants were female. In terms of their regional distribution, 28 % were located in the Southern region, 26 % in both Western and Dublin ML, and 20 % in Dublin NE. With reference to pharmacology, 52 % were prescribed an ‘N’ prescription item, 45 % an ‘A’ item, 39 % an ‘R’ item and 36 % a ‘C’ prescription item. In 2012, 19 % of claimants were prescribed items from ATC groups other than ‘A’ ‘C’ ‘N’ or ‘R’accounting for almost €19 million of the total annual cost.

The following descriptive analysis is based on claimants (*n* = 1,313,825) who were prescribed an ‘N’, ‘C’, ‘A’ or ‘R’ prescription item. Table 2 presents GMS expenditure (€) by claimants categorised by age category and ATC group.

Table 2 shows that 51 % of claimants in the under 5’s were prescribed ‘R’ prescription items, whereas just 2 % were prescribed ‘C’ items. Of those aged 25–34, 42 % were prescribed ‘N’ items, whereas 8 % were prescribed ‘C’ items. For those aged 75 and over, 30 % were prescribed ‘C’ items with 16 % prescribed ‘R’ items. Analysis of expenditure by ATC level 2 identified that respiratory drugs, principally for the treatment of obstructive airway diseases such as asthma, recorded the highest proportion of claimants and total expenditure for those up to the age of 15. Nervous system drugs predominantly for the treatment of depression, anxiety, psychotic conditions, and epilepsy, recorded the highest proportion and expenditure for those between 16 and 64 years. Cardiovascular drugs, particularly statins, recorded the highest proportion and expenditure for those over 65.

**Table 2:** Percentage of GMS Claimants & Expenditure Euro million (€m) by ATC group and Age Category (2012)

Age Cohort	N		C		A		R		Total (€m)	Total GMS
	%	(€m)	%	(€m)	%	(€m)	%	(€m)	N,C,A,R	Cost (€m)
<5	16	0.57	2	0.35	32	0.88	**51**	**2.01**	3.81	12.35
5–11	10	1.78	2	0.29	24	1.13	**64**	**4.33**	7.53	9.46
12–15	28	1.87	2	0.11	20	0.97	**50**	**2.15**	5.10	4.76
16–24	**40**	**9.32**	5	0.52	25	3.44	31	3.91	17.20	17.12
25–34	**42**	**27.11**	8	1.80	26	7.29	24	5.32	41.52	23.69
35–44	**39**	**39.83**	13	6.83	26	12.39	22	7.62	66.68	28.19
45–54	**34**	**46.03**	21	19.31	26	19.79	19	11.32	96.45	35.65
55–64	**29**	**46.33**	27	40.68	26	29.53	18	18.56	135.11	55.41
65–69	26	23.58	**30**	**31.59**	26	19.27	17	13.06	87.49	37.30
70–74	26	27.10	**32**	**43.61**	26	23.28	16	15.22	109.21	51.13
>75	27	79.32	**30**	**93.97**	27	54.78	16	31.85	259.92	119.79
% of Total	36	302.85	29	239.06	21	172.76	14	115.34	830.01	394.86

*N* Nervous System, *C* Cardiovascular System, *A* Alimentary Tract & Metabolism, *R* Respiratory System

The most expensive age category by ATC group is highlighted in bold

Of overall expenditure in 2012, 55 % was due to prescribing claims by females with the proportion of nervous system items (31 %) the major component of this expenditure (Table 3). There was a higher proportion of male claimants up to age 15 in ATC groups ‘R’, and a higher proportion up to age 11 for ATC ‘N’.

**Table 3:** Percentage of GMS Claimants & GMS Expenditure (€m) by ATC group and Sex (2012)

Sex	N		C		A		R		Total (€m)	Total GMS
	%	(€m)	%	(€m)	%	(€m)	%	(€m)	N,C,A,R	Cost (€m)
Females	31	**178.02**	20	**125.31**	27	**96.23**	22	62.80	**462.37**	687.12
Males	29	124.82	22	113.75	25	76.53	23	52.54	367.64	537.74
% of Total	36	302.85	29	239.06	21	172.76	14	115.34	830.01	1224.86

*N* Nervous System, *C* Cardiovascular System, *A* Alimentary Tract & Metabolism, *R* Respiratory System

The most expensive group (females) is highlighted in bold

The underlined data in Table 3 shows the highest prescribing frequency for females (N,A) and males (C,R)

Table 4 shows that the South region contributed over €240 m (29 %) to the overall prescribing expenditure in 2012 and was the most expensive region across the four ATC groups. Drugs for the treatment of the nervous system records the highest proportion across the four regions. Dublin ML and Dublin NE record the highest percentage of claimants for the nervous and respiratory system and the West and South record the highest percentage of cardiovascular claimants. Of the 427,257 claimants in Dublin ML, incorporating Dublin City, 31 % were prescribed nervous system items, whereas just 20 % were prescribed cardiovascular items, largely explained by a relatively younger population in Dublin ML.

**Table 4:** Percentage of GMS Claimants and Expenditure (€m) by ATC group and HSE Region (2012)

Region	N		C		A		R		Total
	%	(€m)	%	(€m)	%	(€m)	%	(€m)	(€m)
Dublin ML	31	79.85	20	61.18	26	46.26	23	31.39	218.67
Dublin NE	31	56.05	21	46.45	26	33.05	23	22.46	158.01
West	30	78.91	22	60.38	26	44.25	22	29.36	212.91
South	30	**88.04**	22	**71.05**	26	**49.20**	22	**32.13**	240.42
% of Total	36	302.85	29	239.06	21	172.76	14	115.34	830.01

*N* Nervous System, *C* Cardiovascular System, *A* Alimentary Tract & Metabolism, *R* Respiratory System

The most expensive group (South) is highlighted in bold

## Results of empirical analysis

In Table 5 the results of the empirical analysis are presented based on estimates from three models. Model 1 shows that whilst age, sex and regions are all significant drivers of expenditure, gender (female) had the greatest impact (β = 0.107). Additional claimants in the South region will have greatest cost increasing impact. However, when the ATC groups were included in model 2, the impact of gender was diluted, with cardiovascular prescribing most influential (β = 1.229) and the explanatory power of the model increases from 40 % to 60 %. The inclusion of age dummies in model 3 expectedly shows those aged over 75 (β =1.195) added most to GMS costs. Multicollinearity was not violated as VIF values did not exceed ten, Tolerance values were greater than 0.1. Mahalanobis and Cook’s distances tests were carried out to check for outliers. For the residuals output, the max value for Cook’s distances did not exceed 1 and the max value for Mahalanobis distances did not exceed the critical χ^2^ value.

**Table 5:** Estimates from Multivariate Regression Models 1–3

Variables	Model 1			Model 2			Model 3		
	β	C1 95 %	t-stat	β	C1 95 %	t-stat	β	CI 95 %	t-stat
Constant	3.339			3.288			3.324		
Sex (F)	0.107	(0.103;0.112)	^a^47.84	0.018	(0.015;0.022)	^a^10.07	0.025	(0.021;0.029)	^a^13.66
Age	0.044	(0.044;0.045)	^a^1043.44	0.015	(0.015;0.015)	^a^306.74			
Dublin ML	0.026	(0.019;0.032)	^a^7.76	−0.003	(−0.008;0.002)	^a^-1.16	−0.003	(−0.009;0.002)	−1.20
West	−0.017	(−0.024;-0.011)	^a^-5.16	0.008	(0.003;0.014)	^a^3.07	0.009	(0.003;0.014)	^a^3.21
South	0.056	(0.050;0.063)	^a^17.08	0.038	(0.033;0.043)	^a^14.21	0.037	(0.032;0.043)	^a^14.00
Alimentary				0.911	(0.907;0.915)	^a^440.64	0.903	(0.899;0.907)	^a^436.51
Cardiovascular				1.229	(1.224;1.234)	^a^478.42	1.193	(1.188;1.198)	^a^452.28
Nervous				0.927	(0.923;0.931)	^a^456.61	0.947	(0.943;0.951)	^a^454.63
<5							0.117	(0.106;0.128)	^a^21.57
5–11							0.103	(0.092;0.113)	^a^19.23
16–24							0.237	(0.227;0.247)	^a^45.55
25–34							0.370	(0.360;0.381)	^a^72.09
35–44							0.475	(0.465;0.485)	^a^92.06
45–54							0.706	(0.696;0.717)	^a^131.29
55–64							0.957	(0.946;0.968)	^a^172.64
65–69							1.071	(1.059;1.083)	^a^173.49
70–74							1.126	(1.114;1.137)	^a^189.07
>75							1.195	(1.184;1.206)	^a^215.44
Adjusted R^2^		0.402		0.604			0.605		

^a^Statistically significant at 5 % level

The dependent variable was the logarithim of annual total GMS cost (€) per claimant

Baseline - Males, age category 12–15, Dublin NE and ATC ‘R’. (least cost variables)

## Discussion

Our investigation identified significant factors [age, sex, region and pharmacology of the drugs] influencing mean GMS expenditure per claimant in Ireland for 2012. This research also identified the drugs (ATC level 5) that incur the highest level of expenditure by age group. The impact of age on expenditure followed a similar pattern as observed in the literature, highest expenditure on respiratory drugs for those under 15, highest on nervous system drugs for those between 16 and 64, and highest on cardiovascular drugs for those aged 65 and over. A recent study reported that children (0–11) and the elderly (70+) will be a key driver of future drug costs in Ireland [3]. A three country study (UK, Italy & Netherlands) found the respiratory system to be one of 3 ATC groups with the highest prevalent rates in children [20]. A study in Ireland found that cardiovascular disease is the most prevalent condition in an elderly population aged 70 and over, indicating the trend has continued between 2004 and 2012 [21]. However, a study in Denmark found treatment intensity and prevalence contributed to drug expenditure costs rather than population aging [22].

In terms of gender differences in prescribing, females account for more than half of GMS claimants in 2012, and were found to be more expensive across the 4 ATC groups with the highest expenditure for nervous system drugs and the smallest for cardiovascular. A study on a Swedish population in 2010 concurs with our findings, which found a greater proportion of men were prescribed cardiovascular drugs whilst a greater proportion of women were prescribed nervous system drugs [23]. A higher female per capita expenditure was reported for nervous and alimentary tract and metabolism drugs in a study in British Colombia with males highest expenditure on cardiovascular drugs [24]. Coronary artery disease was more common in men over 60 than females in Sweden [25]. We reported that males under 11 years were prescribed more prescription items than female which concurs with higher drug usage in adolescent males compared to females in both Catalonia and Sweden [23, 26].

In terms of regional variation, the South region recorded the highest prescribing expenditure and Dublin NE the lowest. Dublin ML recorded the highest proportion of nervous prescription items and Dublin ML recorded the lowest for cardiovascular items. Two Irish studies [27, 28] found variations for Ischaemic heart disease and diabetes respectively, in prescribing by old health board regions.^4^ They found high prescribing rates in the Midlands and Eastern regions and low prescribing rates in the Western and North-Western regions. Furthermore, an Irish study found variation in projected drug costs by old health board regions where the Midlands region was the most expensive region which is now part of the Dublin ML region [3]. Research on prescribing in Canada reported that individual characteristics and living environment affect prescription use and may explain regional variations in prescription use. For example, area level measures of population health and socioeconomic status affected the likelihood of prescription use [29].

In examining the factors driving expenditure, we also investigated the most costly prescription items. Drugs for the treatment of the nervous system were the greatest contributor to prescribing expenditure in 2012. An important finding of this paper identified the most expensive drugs in each ATC group by age cohort. Escitalopram which treats depression and anxiety accounts for the highest expenditure (€0.74 m; 1 % of ‘N’) for 16 to 24 year olds and Olanzapine which treats psychotic conditions such as schizophrenia and bipolar disorder accounts for the highest expenditure (€2.48 m; 1 % of ‘N’) for 25 to 34 year olds. Expenditure on Pregabalin (€16.95 m; 10 % of ‘N’) in 35 to 64 year olds for the treatment of conditions such as epilepsy contributed to 10 % of prescription items for ATC ‘N’.

Respiratory drugs was the main contributor to GMS drug expenditures in children up to the age of 15 and this is corroborated by the literature [26, 30]. With an increasing incidence of asthma in developed countries, it’s not surprising that drugs for the treatment of obstructive airway diseases, such as Montelukast (€2.4 m; 29 % of ‘R’), and inhalers such as Salbutamol and Beclometasone are amongst the greatest contributors to expenditure for ATC group ‘R’. A childhood study in Ireland reported that cigarette smoke, atopy, and the presence of certain furry pets, are determinants of respiratory symptoms in children [31].

Cardiovascular prescription expenditure in 2012 was mainly influenced by the prescribing of statins (Atorvastatin, Rosuvastatin, Pravastatin, Simvastatin and Fluvastatin) [7]. Atorvastatin has since come off patent, which should help reduce costs associated with high cholesterol. The greater promotion of generic prescribing for these drugs would lead to cost savings.

Expenditure on Esomeprazole (€31.25 m; 18 % of ‘A’), a Proton Pump Inhibitor (PPI) which is used to treat certain stomach and oesophagus conditions, such as ulcers and acid reflux, contribted 18 % of expenditure on alimentary tract and metabolism items. In 2012, PPI’s were the second most expensive group of medicines after statins [7]. An Australian study investigated the utilisation of PPI’s and found Esomeprazole to be the second most prescribed PPI and the second most expensive group of medicines after statins [32]. An Irish study on the GMS scheme investigated whether cost savings could be made by changes in prescribing practice. They found if Esomeprazole was substituted with a less expensive alternative, it would create savings of approximately €3.3 million [33].

Our analysis highlighted a significant difference between the mean expenditure per claimant and the median. This is explained by high cost users of prescription items in our population. The characteristics of high cost users need further investigation. An important strength of this study is that it provides a population based overview of drug utilisation and expenditure across four ATC groups.

This study is subject to certain limitations. The HSE-PCRS has pre-defined age cohorts, therefore making it difficult to re-construct age cohorts to make comparisons. The age band widths are not equal across the 11 HSE age cohorts. The HSE-PCRS database lacks a unique health identifier, which precludes the link between pharmacy data and other health data such as mortality data. The analysis is limited to the HSE-PCRS database which relates to public patients only.

## Conclusion

All European countries have common concerns about growing drug expenditures. The growing proportion of the elderly, the increase in the incidence and duration of chronic diseases, the continuing development of health technologies, and the increase in health expectation of society, are common factors across all countries. Our research indicates that growth in the proportion of elderly claimants and associated levels of cardiovascular prescribing, particularly for statins, will present difficulties for Ireland in terms of cost containment. The primary prevention of CVD is dependent on the effective reduction of the major risk factors, particularly tobacco control and a healthier diet. Policies aimed at population-wide CVD prevention, such as legislating for smoke-free public spaces, banning dietary transfats, or halving daily dietary salt intake are generally effective and cost saving. Based on prevalence rates and related prescribing costs of asthma among children up to the age of 15, it is imperative that public health resources and educational efforts are targeted to improve asthma control and reduce the disease burden on both the healthcare system and society. Expenditure on nervous system drugs in Ireland are highest for those aged between 16 and 64, in particular, drugs for the treatment of anxiety and depression. The treatment of anxiety and depression is vitally important with the growth in suicidal related deaths in Ireland. Whilst pharmacological treatments are often necessary, changes in prescribing practice combined with psychological therapies, cognitive methods, and self-help strategies, can yield cost savings. Since October 2013, a generic substitute for Esomeprazole is now available which could generate substantial savings in alimentary tract prescribing. Whilst policies aimed at cost containment such as co-payment charges, generic substitution, reference pricing, and adjustments to GMS eligibility can be used to curtail expenditure, health promotional programs and educational interventions should be given equal emphasis.

Furthermore, policies intended to affect physicians’ prescribing behaviour include guidelines, information (about price and less expensive alternatives) and feedback, and the use of budgetary restrictions could also yield significant savings.

## Abbreviations

A: Alimentary
ATC: Anatomical Therapeutic Chemical
C: Cardiovascular
CDS: Community Drug Schemes
CVD: Cardiovascular Disease
EU: European Union
G: Genito urinary system & sex hormones
GDP: Gross Domestic Product
GMS: General Medical Services
HSE: Health Service Executive
J: Anit-infectives for systemic use
ML: Mid-Leinster
N: Nervous
NE: North-East
OECD: Organisation for Economic Co-operation and Development
OLS: Ordinary Least Squares
PCRS: Primary Care Reimbursement Service
PPI: Proton Pump Inhibitor
R: Respiratory
VAT: Value Added Tax
VIF: Variance Inflation Factor
WHO: World Health Organisation

## Footnotes

1 A claimant is a person who is eligible for a GMS card and makes a claim.

2 GMS coverage refers to the proportion of the population who have a medical card.

3 In the Anatomical Therapeutic Chemical (ATC) classification system, the active substances are divided into different groups according to the organ or system on which they act and their therapeutic, pharmacological and chemical properties. Drugs are classified in groups at five different levels. The drugs are divided into fourteen main groups (1^st^ level), with pharmacological/therapeutic subgroups (2^nd^ level).

4 The 8 Health Service Executive (HSE) regions were restructured to 4 Regions (Dublin ML; Dublin NE; South; West) in 2010.
